# Antimicrobial Resistance Gene Delivery in Animal Feeds

**DOI:** 10.3201/eid1004.030506

**Published:** 2004-04

**Authors:** Karen Lu, Rumi Asano, Julian Davies

**Affiliations:** * University of British Columbia, Vancouver, B.C., Canada; † University of California, Berkeley, California, USA

**Keywords:** antimicrobial agents, resistance, therapeutic, prophylactic, agriculture, enterococci

## Abstract

Avoparcin, a glycopeptide antimicrobial agent related to vancomycin, has been used extensively as a growth promoter in animal feeds for more than 2 decades, and evidence has shown that such use contributed to the development of vancomycin-resistant enterococci. A cluster that includes three genes, *vanH, vanA, and vanX,* is required for high-level resistance to glycopeptides. In the vancomycin producer *Amycolatopsis orientalis* C329.2, homologs of these genes are present, suggesting an origin for the cluster. We found substantial bacterial DNA contamination in animal-feed-grade avoparcin*.* Furthermore, nucleotide sequences related to the cluster *vanHAX* are present in this DNA, suggesting that the prolonged use of avoparcin in agriculture led to the uptake of glycopeptide resistance genes by animal commensal bacteria, which were subsequently transferred to humans.

Antimicrobial resistance in bacterial pathogens is a major impediment to successful therapy, and in several instances, bacterial strains have arisen that are refractory to most available antimicrobial treatments (*1*). Resistance arises by mutation (influencing the target or efflux of the antimicrobial agent) or by the acquisition of resistance genes (encoding antimicrobial or target alteration, or alternate pathways) (*2*,*3*). The actual origins of acquired resistance genes are unknown, but environmental microbes, including the strains producing antimicrobial agents, are believed to be important sources (*4*,*5*). Substantial genetic and biochemical similarities exist between resistance determinants in antimicrobial agent–producing actinomycetes and resistance genes found in gram-positive and gram-negative pathogens (*6*–*9*).

Since vancomycin-resistant enterococci (VRE) were clinically isolated in Europe (1986) and the United States (1987), VRE infections have been reported throughout the world. These infections may be life-threatening because choices for alternative treatment are limited. Concomitant with human use of vancomycin, avoparcin, a closely related glycopeptide antimicrobial agent, has been widely used in Europe and other continents as an animal growth promoter (Figure 1). VRE have been isolated, commonly from pigs and chickens fed avoparcin-containing animal feed**,** and humans coming into contact with the animals (farm workers, butchers) have been shown to carry VRE (*10*–*12*); identical clones have been found (*13*). The public health concern about the emergence and dissemination of VRE in food animals and the food supply caused the European Union to ban the use of avoparcin in animal feed in 1997. The discontinued use of avoparcin in animal feed has resulted in a reduction in the number of vancomycin-resistant organisms isolated from animals (*14*,*15*).

**Figure 1:**
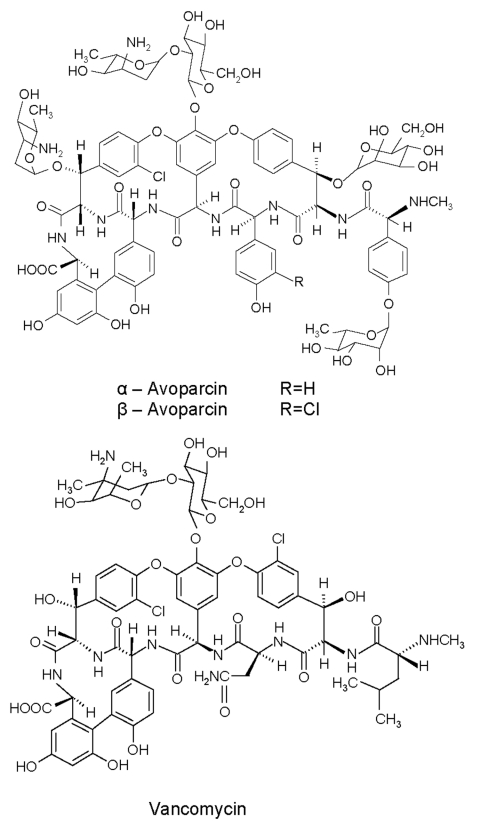
Chemical structures of avoparcin and vancomycin.

High-level glycopeptide resistance is conferred by a cluster of three genes, *vanH, vanA, vanX* (the *van* cluster), plus associated regulatory elements; the cluster is often carried by conjugative transposons (*16*–*18*). The *vanH* gene encodes a D‑lactate dehydrogenase that provides the requisite D‑lactate. *vanX* encodes a highly specific DD-peptidase that cleaves only D-Ala-D-Ala produced endogenously while leaving D-Ala-D-Lac intact. The third gene, *vanA*, encodes an ATP-dependent D-Ala-D-Lac ligase. Replacement of D-Ala-D-Ala by D-Ala-D-Lac in the bacterial cell wall results in a thousandfold reduction in the binding of glycopeptide antimicrobial agents to their peptidoglycan target (*19*). Studies have demonstrated the presence of *vanHAX* homologs, such as *vanH-ddlN-vanX* (Figure 2), in actinomycete strains producing glycopeptides, and strong structural and functional similarity exists between the various homologs and the *van* cluster of VRE (*8*,*9*). Some researchers have proposed that the *vanH*, *vanA*, and *vanX* genes of hospital enterococci may have been acquired en bloc from the actinomycetes (*8*). Related *vanHAX* gene clusters have been identified in *Paenibacillus* spp. by Patel and coworkers, indicating another possible source of the *van* cluster (*20*). Regardless of the microbial source, the feeding of crude antimicrobial preparations to animals is plausible as a delivery process for transferring the cognate antimicrobial resistance genes between producing strains and the commensal bacteria of animals (*21*); the concomitant selection for resistance would ensure the survival of rare resistant strains. We provide evidence that a DNA-encoding homolog of the *van* cluster is a contaminant of feed-grade avoparcin and propose that animal use both created and selected for glycopeptide-resistant strains. The emergence of vancomycin-resistant *Staphylococcus aureus* (VRSA) is a recent sequela to this train of events involving the *van* gene clusters (*22*).

**Figure 2:**
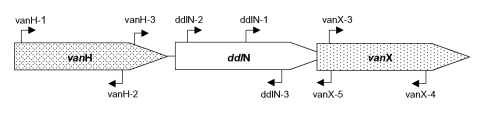
The *vanHAX* cluster of *Amycolatopsis coloradensis* NRRL 3218.

## Materials and Methods

### DNA Extraction from Avoparcin

A suspension (0.7 mL) of avoparcin (Roche, Sydney, Australia) in H_2_O (100 μg/mL) was centrifuged in a 1.5-mL Eppendorf tube for 6 min and the supernatant, after being shaken with 1 volume of phenol:chloroform:isoamyl alcohol (25:24:1), was centrifuged at 16,000 x *g* for 3 min. The aqueous phase was subjected to two additional phenol-chloroform extractions. The nucleic acid in the pooled aqueous fractions was precipitated with ethanol; the pellet was recovered by centrifugation and further purified by using a GeneClean spin kit (BIO101) and resuspended in 100 μL of double-distilled H_2_O. The DNA concentration was measured with a fluorometer (Model TKO100, Hoefer Scientific Instruments, San Francisco, CA).

### PCR Amplification of 16S rDNA Sequences

Primers 16S 440F and 16S 1491R (Table) were designed to amplify partial 16S rDNA sequences. The polymerase chain reaction ((PCR) contained 2 mM MgCl_2_, 0.16 mM dNTP, 0.4 μL of each primer, *Taq* polymerase (1 U), 3‑15 ng template, and 5% dimethyl sulfoxide (DMSO). PCR was done in a MiniCycler (MJ Research, Waltham, MA) by using the following program: 96°C, 3 min; 96°C, 30 s; 60°C, 45 s; 72°C, 1 min 30 s; 35 cycles; and 72°C, 10 min.

**Table:** Primer sequences for genes *vanH*, *ddlN*, *vanX* and partial 16S rDNA

Primer	Primer sequences
vanH-1	5′-CAC ATC GA(C/T) GTG GAA TAC GC-3′
vanH-2	5′-CAG TCG GCG TAG AAG ATG CC-3′
vanH-3	5′-GAG GAA GGC ATC TTC TAC GC-3′
ddlN-1	5′-ACG (G/C)CA GTA CGA C(G/T)C GAA G-3′
ddlN-2	5′-T(G/T)C CTG GA(A/T) GCT (G/C)TG CGA C-3′
ddlN-3	5′-G(A/G)T AAC GGC TGT ACG AGG TC-3′
vanX-3	5′-CCA CGT GGG ACA ACT TCA C-3′
vanX-4	5′-CAG (C/G)(G/T)T GTA GTG CCA CCA CTC-3′
vanX-5	5-TCA CCA GAT ATC CGT CCA CC-3′
16S 440F	5′-AGC AGG GAA GAA GCG (A/T/C)(A/G)A GT-3′
16S 1491R	5′-CGG CTA CCT TGT TAC GAC TTC-3′

### PCR Amplification of *vanH, ddlN,* and *vanX* Sequences

Different combinations of PCR primers (*9*) were used to amplify the entire *van* cluster (Table). Reaction conditions were as described previously.

### Cloning of *vanH, ddlN, vanX,* and Partial 16S rDNA Genes

PCR products were cloned by using vector pCR 2.1-TOPO (Invitrogen, Burling, Ontario, Canada) according to the manufacturer’s instructions, and the insertion size was confirmed by a second PCR. Plasmid DNA was extracted by using the Concert rapid plasmid miniprep system (Invitrogen).

### DNA Sequence Analysis

Cycle sequence reactions were carried out with a BigDye terminator DNA sequencing kit (Applied Biosystems, Foster City, CA) with plasmid DNA templates. The cycle sequence program was as follows: 96°C, 1 min; 96°C, 30 s; 50–60°C (dependent on different primers and fragments), 15 s; 60°C, 4 min, for 25 cycles. Excess oligos and dyes were removed by using CentriSep spin columns (Princeton Separations, Aldelphia, NJ). Reaction products were sequenced by the Nucleic Acid and Protein Service, University of British Columbia, using an ABI PRISM 377 sequencer. Sequences were analyzed by using the standard nucleotide-nucleotide BLAST program (National Center for Biotechnology Information, Bethesda, MD). and comparisons were carried out by using CLUSTAL W (European Bioinformatics Institute, Cambridge, UK).

## Results and Discussion

**Figure 3:**
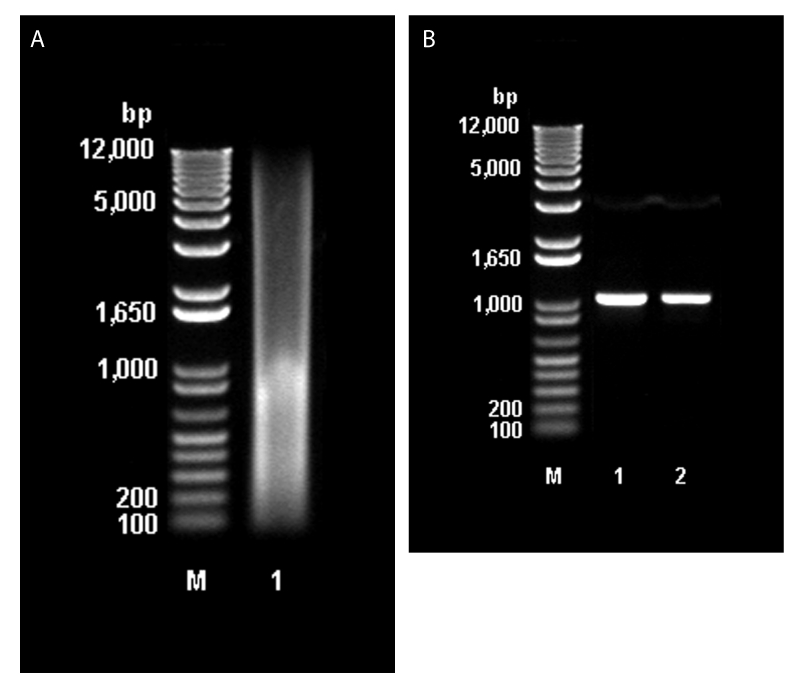
A, Molecular mass of total DNA extracted from animal feed grade avoparcin. M: 1 kb plus DNA ladder (Invitrogen, Burlington, Ontario, Canada). Lane 1: sample of total DNA extracted from animal feed–grade avoparcin. B, Polymerase chain reaction amplification of partial 16S rDNA (1051 bp) with primers 16S 440F and 16S 1491R. M:1 kb plus DNA ladder. Lane 1: DNA extracted from animal feed grade avoparcin. Lane 2: DNA of the avoparcin producer *Amycolatopsis coloradensis* NRRL 3218.

Direct extraction of avoparcin powder with phenol/chloroform/isoamyl alcohol provided substantial amounts of DNA (30.5 μg/g of avoparcin) (Figure 3A). PCR amplification of the DNA with oligonucleotide primers specific for a region of streptomycete 16S rRNA gave a single amplicon (Figure 3B), which was sequenced and shown to be 16S closely related to that of *Amycolatopsis coloradensis*, the producer of avoparcin. Figure 3B shows similarities between the 16S rRNA of species that produce glycopeptide antimicrobial agents.

**Figure 4:**
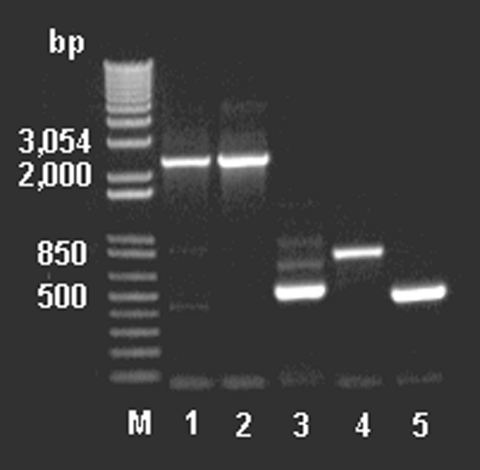
Polymerase chain reaction amplification of the partial *vanHAX* clusters from DNA extracted from animal feed–grade avoparcin and the antibiotic producer *Amycolatopsis coloradensis* NRRL 3218, and genes *vanH, ddlN,* and *vanX* from DNA extracted from animal feed grade avoparcin. M: 1 kb plus DNA ladder. Lanes 1–2: the partial *van* cluster (2.3–2.4 kb) amplified with primers vanH‑1 and vanX‑4; lane 1: DNA extracted from animal feed grade avoparcin; lane 2: DNA of the avoparcin producer *A. coloradensis* NRRL 3218. Lane 3: *vanH* (500 bp) amplified with primers vanH‑1 and vanH‑2. Lane 4: *ddlN* (850 bp) amplified with primers ddlN‑2 and ddlN‑3. Lane 5: *vanX* (500 bp) amplified with primers vanX‑3 and vanX‑4.

To examine for the presence of genes involved in glycopeptide resistance from the antimicrobial agent–derived DNA, we used the DNA primers described by Marshall et al. (*9*). The amplicons (Figure 4) were cloned, sequenced, and assembled, indicating a *van*-like cluster closely related to that found in *A. orientalis* and *Streptomyces toyocaensis*. Control reactions run without added template were negative.

The genes encoded three putative proteins showing >50% amino acid identity to the Van H, A, and X proteins of VRE (Figure 2). All of the clusters have translational overlaps between the *vanA* and *vanX* genes and their homologs, suggesting cotranslational regulation of expression. This finding clearly implies that the *van* cluster must be transferred and acquired in toto from any source organism.

We suggest that the use of crude avoparcin preparations in animal feeds from 1975 to 1996 was the origin of the *vanHAX* cluster in the genesis of VRE (and possibly that found in VRSA) (*22*,*23*). Large amounts of avoparcin were used in animal feed; in Denmark, for example, total vancomycin use in 1994 amounted to 24 kg, whereas avoparcin use in animals was 24,000 kg (*24*). During their entire lives, broiler chickens received 15 mg/kg and pigs 20–40 mg/kg of antimicrobial agentin their feed. Each pig was fed 5–10 g of the crude drug for its life span and, consequently, received a steady dose of DNA encoding vancomycin resistance. In Europe, an estimated 100 mg of antimicrobial agents are used in animal feed for the production of 1 kg of meat for human consumption. We believe that this regimen would have favored the selection and maintenance of rare bacterial transformants carrying the resistance genes. If one bears in mind that large numbers of pigs and chickens were exposed to the antimicrobial agent, the probability of gene pick-up by bacterial commensals in the animal gastrointestinal tract would be favored, and once incorporated into a gut commensal genome, further dissemination would have followed under antimicrobial selection. The finding that organization of the *van* cluster in contaminating DNA of the feed is identical to that in VRE, with overlapping reading frames typical of translational coupling of gene expression between the *vanA* and *vanX* homologs (*9*), reinforces this supposition.

The mechanism by which a *van* cluster becomes functionally integrated into bacteria is not known. We propose that intestinal bacteria were the original recipients of the DNA; many of the resident strains are known to be competent for DNA uptake (*25*,*26*). However, mere uptake is not sufficient for function, and the actinomycete genes differ from VRE genes in their G+C content (approximately 65% vs. 50%) and codon usage. Given the enormous complexity of bacterial populations in the mammalian gastrointestinal tract (*27**,**28*), we assume that a variety of intestinal species may have incorporated the resistance-encoding DNA; expression (at low levels) would have been rare, depending on the compatibility of the *van* genes with the transcription and translation system of the host. Under constant antimicrobial selection pressure, translationally competent sequences would have developed by mutation; this would not necessarily have occurred in enterococci. Nonetheless, the conversion (evolution) of the actinomycete genes into functional enterococcal genes likely would have required many generations of growth under constant selection, and any intermediate stages in this process are a matter of speculation; however, once established on conjugative transposons, the genes would be readily disseminated (*17*,*29*). A number of similar *van* clusters have been identified in different bacterial species, and whether these evolved independently or by divergent evolution is unknown.

The finding of resistance genes in crude antimicrobial products intended to be fed to animals adds to the already strongly voiced opinion that use of antimicrobial agents in this way constitutes a serious public health concern and further emphasizes the need for prohibiting the use in animal feed of all antimicrobial agents that are employed in human therapy. This ban should include structurally or biologically related antimicrobial agents and the use of any compound with the potential to select for cross-resistance to another antimicrobial agent (*15*,*30*). The use of avoparcin in Denmark was prohibited in 1995 and in the European Union in 1997. Subsequently, several other antimicrobial growth promoters were banned (*31*,*32*). However, the United States and Canada permit the use of many such products, including penicillin, tetracycline, macrolides, and sulfonamides**.** Nonhuman applications of antimicrobial agents, such as in agriculture and aquaculture, should employ only chemically and biologically distinct classes of compounds developed specifically for that purpose*.* Clearly such measures should be combined with a requirement for rational and prudent measures for antimicrobial use in the human population.

Many antimicrobial agents (or their close structural relatives) have been used extensively as animal-feed additives. In almost all cases, crude antimicrobial preparations are used, and thus the antimicrobial agent acts as a carrier for its cognate resistance genes. These delivery systems provide the opportunity for resistant strains of bacteria to evolve and so create an enormous gene pool for antimicrobial resistance determinants in the environment.
